# Moderate activation of IKK2-NF-kB in unstressed adult mouse liver induces cytoprotective genes and lipogenesis without apparent signs of inflammation or fibrosis

**DOI:** 10.1186/s12876-015-0325-z

**Published:** 2015-07-30

**Authors:** Hong Lu, Xiaohong Lei, Qinghao Zhang

**Affiliations:** Department of Pharmacology, SUNY Upstate Medical University, 750 E Adams ST, Syracuse, NY 13210 USA

**Keywords:** IKK2, IKK1, NF-kB, RelB, Lxr, Liver, Mice, Lipogenesis, Inflammation, Fibrosis

## Abstract

**Background:**

The NF-kB signaling, regulated by IKK1-p52/RelB and IKK2-p65, is activated by various stresses to protect or damage the liver, in context-specific manners. Two previous studies of liver-specific expression of constitutive active IKK2 (IKK2ca) showed that strong activation of IKK2-NF-kB in mouse livers caused inflammation, insulin resistance, and/or fibrosis. The purpose of this study was to understand how moderate activation of IKK2-NF-kB in adult mouse livers alters hepatic gene expression and pathophysiology.

**Method:**

We generated mice with adult hepatocyte-specific activation of Ikk2 (Liv-Ikk2ca) using Alb-cre mice and Ikk2ca Rosa26 knockin mice in which a moderate expression of Ikk2ca transgene was driven by the endogenous Rosa26 promoter.

**Results:**

Surprisingly, compared to wild-type mice, adult male Liv-Ikk2ca mice had higher hepatic mRNA expression of Ikk2 and classical NF-kB targets (e.g. Lcn2 and A20), as well as IKK1, NIK, and RelB, but no changes in markers of inflammation or fibrosis. Blood levels of IL-6 and MCP-1 remained unchanged, and histology analysis showed a lack of injury or infiltration of inflammatory cells in livers of Liv-Ikk2ca mice. Moreover, Liv-Ikk2ca mice had lower mRNA expression of prooxidative enzymes Cyp2e1 and Cyp4a14, higher expression of antioxidative enzymes Sod2, Gpx1, and Nqo1, without changes in key enzymes for fatty acid oxidation, glucose utilization, or gluconeogenesis. In parallel, Liv-Ikk2ca mice and wild-type mice had similar levels of hepatic reduced glutathione, endogenous reactive oxygen species, and lipid peroxidation. Additionally, Liv-Ikk2ca mice had higher Cyp3a11 without down-regulation of most drug processing genes. Regarding nuclear proteins of NF-kB subunits, Liv-Ikk2ca mice had moderately higher p65 and p50 but much higher RelB. Results of ChIP-qPCR showed that the binding of p50 to multiple NF-kB-target genes was markedly increased in Liv-Ikk2ca mice. Additionally, Liv-Ikk2ca mice had moderate increase in triglycerides in liver, which was associated with higher lipogenic factors Pparγ, Lxr, Fasn, Scd1, and CD36.

**Conclusion:**

In summary, moderate activation of IKK2-NF-kB in unstressed adult mouse hepatocytes produces a cytoprotective gene expression profile and induces lipogenesis without apparent signs of inflammation or fibrosis, likely due to strong activation of the anti-inflammatory IKK1-RelB alternative NF-kB pathway as well as the Lxr.

**Electronic supplementary material:**

The online version of this article (doi:10.1186/s12876-015-0325-z) contains supplementary material, which is available to authorized users.

## Background

Oxidative stress and inflammation are highly prevalent in major liver diseases, such as viral hepatitis, alcoholic and non-alcoholic steatohepatitis, liver cirrhosis, and liver cancer. Being a master regulator of inflammation and cytoprotection [1], NF-kB signaling is tightly regulated by the inhibitor of nuclear factor-kappaB (IkappaB) kinase 1 (IKK1) and IKK2. In unstimulated cells, NF-kB dimers p65 and p50 are sequestered in cytosol by inhibitor of kB α (IkBα). The IKK complex has two catalytic subunits, IKK2 and IKK1, which activate NF-kB via the classical and alternative pathways, respectively [2]. In classical pathway, upon activation by stimuli such as tumor necrosis factor α (TNFα), IKK2 is phosphorylated, which in turn phosphorylates IkBα. This leads to degradation of IkBα, nuclear translocation of p65/p50, and induction of NF-kB–dependent genes, including inflammatory genes TNFα and interleukin-6 (IL-6). The alternative NF-kB pathway is activated by distinct ligands via phosphorylation of IKK1, leading to processing of p100 and release of p52 and RelB into nucleus, resulting in induction of unique subsets of NF-kB–dependent genes by RelB/p52 and RelB/p50 dimers [1, 2].

NF-κB is a survival factor for hepatocytes [3, 4], and NF-kB is activated by various stresses to protect or damage the liver, in context-specific manners [3]. Thus, to improve the efficacy and decrease the hepatotoxicity of drug treatment, it is of paramount importance to understand the context-specific roles of IKK2-NF-kB signaling in liver pathophysiology, particularly in hepatocytes. Intriguingly, activation of IKK2-NF-kB causes context-specific effects on liver pathophysiology. Activation of IKK2-NF-kB in hepatocytes is critical in protecting hepatocytes from TNFα- and bile-acid-induced cell death [3, 4]. In contrast, superactivation of IKK2 in neonatal liver through Tet-Off hepatocyte-specific transgenic expression of constitutively active IKK2 (IKK2ca) markedly activates p65 and induces proinflammatory genes, chronic inflammation, and liver fibrosis in adult mice [5]. Conversely, when hepatocyte-specific expression of Ikk2ca was driven by a strong albumin promoter, adult Ikk2ca transgenic mice have normal liver histology (no fibrosis) but induction of proinflammatory cytokines IL-1β and IL-6 as well as 2 rate-limiting enzymes for gluconeogenesis, namely phospho-enolpyruvate carboxykinase (Pepck) and glucose-6-phosphatase (G6pc) [6]. In these Ikk2ca transgenic mice, hepatocytes is the primary source of IL-6 production, which plays a key role in insulin resistance [6]. Currently, the mechanism of context-specific roles of IKK2 in hepatic NF-kB activation, gene expression, and pathophysiology remains unknown.

In view of the pathological importance of NF-kB in inflammation and carcinogenesis, NF-kB inhibitors, particularly inhibitors of IKK2 are under active development for treatment of inflammatory diseases and cancer [7]. Drug-induced liver injury is a leading cause of failed clinical trials and withdrawal of FDA-approved drugs from the market [8]. Thus, it is important to understand the role of IKK2-NF-kB in the regulation of genes important in inflammation, nutrient homeostasis, drug metabolism, and liver protection. The purpose of this study was to understand how moderate activation of IKK2-NF-kB in adult liver alters gene expression and pathophysiology. Surprisingly, adult male mice with adult-hepatocyte-specific activation of Ikk2 (Liv-Ikk2ca) had moderate increase in hepatic triglycerides as well as induction of certain classical NF-kB targets and important cytoprotective genes, but no changes in major markers of inflammation or fibrosis. Further study suggested that strong activation of IKK1-RelB, the anti-inflammatory alternative NF-kB pathway, as well as activation of liver X receptor (LXR), a nuclear receptor that promotes lipogenesis but exerts anti-inflammatory effects, might be the underlying mechanism of hepatic induction of cytoprotective genes and lipogenesis without apparent inflammation or fibrosis in these Liv-Ikk2ca mice.

## Methods

### Generation of mice with adult hepatocyte-specific expression of constitutive active Ikk2 (Liv-Ikk2ca)

The R26Stop^FL^ikk2ca mice (Stock # 008242, Jackson Laboratory) have a *loxP*-flanked STOP cassette that prevents the transcription of Ikk2ca from the endogenous Rosa26 gene locus [9]. Liv-Ikk2ca mice (Ikk2ca fl/+, Alb-cre/+) and wild-type littermates (Ikk2ca fl/+, Alb-cre/-) were generated by crossing R26Stop^FL^ikk2ca mice with Alb-cre mice (Stock # 003574, Jackson Laboratory). In Liv-Ikk2ca mice, the removal of the STOP cassette by liver-specific expression of the Cre recombinase allows liver-specific expression of the Ikk2ca transgene driven by the endogenous Rosa26 promoter. Mice were fed rodent chow and allowed water and feed *ad libitum*. Liver and blood samples were collected from adult (3-month old) male Liv-Ikk2ca mice and the wild-type littermates (*N* = 6 per group). Liver tissues were snap frozen in liquid nitrogen upon collection and stored at −80 °C until use. To prepare serum samples, the clotted blood samples were centrifuged at 6000 rpm for 10 min and the resultant supernatants were stored at −80 °C until use. All animals received humane care and all animal procedures in this study were approved by the Institutional Animal Care and Use Committee (IACUC) of the SUNY Upstate Medical University.

### RNA isolation and real-time PCR quantification of mRNA

Total RNA from liver tissues was extracted by using RNA STAT-60 (Tel-Test, Friendswood, TX, USA). cDNA was produced by the use of High Capacity cDNA Reverse Transcription Kit (Applied Biosystems, Foster City, CA, USA) according to the manufacturer’s instructions. The resultant cDNA was used for real-time PCR quantification of mRNA using iQ™ SYBR® Green Supermix (Bio-Rad, Hercules, CA, USA) by MyiQ2™ Two-Color Real-Time PCR Detection System (Bio-Rad). The amount of mRNA was calculated using the comparative CT method, which determines the amount of target normalized to an endogenous reference, β-actin, with values of wild-type set as 1.0.

### Western blot quantification of liver nuclear and cytosolic proteins

Liver nuclear extracts were prepared with a nuclear extract kit (Marligen Biosciences, Inc., Rockville, MD). Liver lysates were prepared by homogenization of liver samples with RIPA buffer. Proteins in the liver nuclear extracts or lysates were resolved in sodium dodecyl sulphate-polyacrylamide gel electrophoresis. Western blot quantification of Ikk1, Ikk2, NF-kB subunits, and Foxo1 in liver nuclear and cytosolic extracts was carried out with the primary antibodies as follows: NF-кB p50 (sc-1190), p52 (sc-298), p65 (sc-109) and RelB (sc-226) from Santa cruz; IKK1 (#2682), IKK2 (#2370), Foxo1 (#2880P) and histone H3 (#4499) from Cell Signaling Technology, Inc; Glyceraldehyde 3-phosphate dehydrogenase (Gapdh) (HPA040067, Sigma-Aldrich). Western blot quantification of Scd1 in liver lysates was carried out with the primary antibody against Scd1 (#2794S, Cell Signaling Technology). Primary antibodies were revealed with HRP-conjugated secondary antibodies (anti-rabbit IgG (W4011) or anti-goat IgG (V805A) from Promega) and ECL Western Blotting Substrate (W1015, Promega). ChemiDoc™ XRS+ System (Bio-Rad) and Image Lab 4.0 software (Bio-Rad) were used to capture signals and determine signal intensities.

### Determination of DNA-binding of NF-kB p50 by chromatin immunoprecipitation quantitative PCR (ChIP-qPCR)

The binding of NF-kB p50 subunit to DNA in mouse liver was quantified by ChIP-qPCR [10]. Briefly, chromatins (5 μg) from livers of wildtype and Liv-Ikk2ca mice were used in ChIP assay using antibodies (4 μg) against NF-кB p50 (sc-1190). The ChIPed DNA fragments were quantified by Qubit^™^ DNA assay kit (Life Technologies), and the enrichment of DNA fragments that contain putative NF-kB binding sites in the gene promoter was quantified by qPCR reaction containing DNA fragments, 500 nM primers (sequences in Additional file 1) and iQ™ SYBR® Green Supermix by MyiQ2™ Two-Color Real-Time PCR Detection System.

### Determination of serum levels of IL-6 and MCP-1

Serum levels of IL-6 and Mcp-1 in wild-type and Liv-Ikk2ca mice were quantified by Bio-Rad Bio-Plex Mouse Cytokine Group I 2-plex Assay kit using the BioRad BioPlex 200 Luminex Instrument. Serum levels of IL-6 and Mcp-1 were calculated with standard curves of IL-6 and Mcp-1 supplied in the kit.

### NAD(P)H quinone oxidoreductase 1 (Nqo1) activity assay

Liver homogenates were prepared in 25 mM Tris–HCl (pH 7.4) containing 250 mM sucrose and 5 μM FAD^+^. After centrifugation of homogenates at 15,000 g, 10 μg of supernatant proteins were used to determine Nqo1 activity as previously described using DCPIP as a substrate [11]. Nqo1 activity is described as the dicumarol inhibitable decrease in absorbance at 600 nm, with values of wild-type group set as 1.0.

### Determination of lipids in mouse liver and serum

Lipids from frozen liver tissue were prepared as described previously [12]. The lipid pellets were dissolved in a mixture of 270 μl of isopropanol and 30 μl of Triton X-100. Triglycerides (TG) and total cholesterol (CHO) in liver and serum were determined using commercial triglyceride and cholesterol analytical kits with standards (Pointe Scientific, Canton, MI).

### Quantification of reduced glutathione (GSH), endogenous reactive oxygen species (ROS), lipid peroxidation, and hydroxyproline in mouse liver

Liver homogenates were used to quantify GSH by Ellman’s reagent and a GSH standard curve [13]. Hepatic levels of endogenous ROS were determined using 2′,7′-dichlorofluorescin diacetate as the fluorogenic probe and a standard curve of 2′,7′-dichlorofluorescein [14]. Lipid peroxidation was determined by quantifying thiobarbituric acid reactive substances (TBARS) using a standard curve of 1,1,3,3-Tetramethoxypropane. To determine liver fibrosis, hepatic levels of hydroxyproline were quantified following an improved method using Chloromine-T reagent and Ehrlich’s reagent as well as a standard curve of hydroxyproline [15].

### Histopathological staining and analysis

Liver samples were preserved in neutral buffered formalin (10 %) before use. Formalin-fixed tissues were embedded in paraffin, sectioned (5 μm), and stained with hematoxylin and eosin (H&E). H&E stained liver sections were evaluated by light microscopy at 200 X magnification for evidence of hepatocyte apoptosis, inflammation, and fibrosis using a Leica DMI3000 B Inverted Light Microscope System with Leica DFC450 C Digital Microscope Camera (Leica Microsystems, Inc.).

### Statistical analysis

All values are expressed as mean ± S.E. The student’s *t*-test was used to determine the statistical difference between Liv-Ikk2ca and wild-type samples (SigmaPlot 12.5). Statistical significance was set at *p* < 0.05.

## Results

### Hepatic mRNA expression of genes important in NF-kB activation, inflammation, and fibrosis in Liv-Ikk2ca mice

**Fig. 1:**
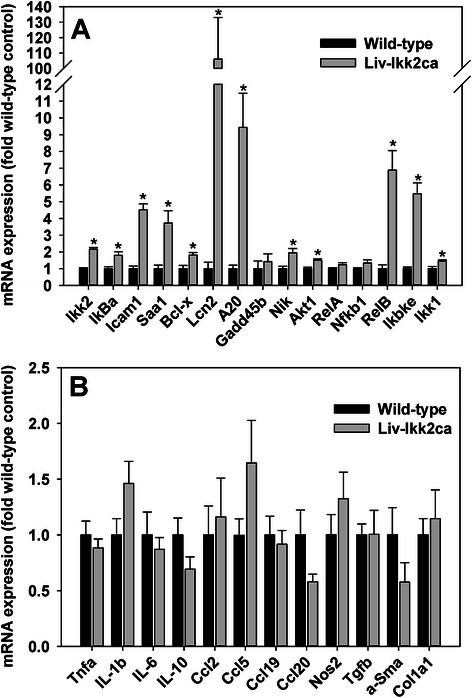
Hepatic mRNA expression of genes important in the IKK-NF-kB pathways, inflammation and fibrosis in adult male mice with hepatocyte-specific activation of Ikk2 (Liv-Ikk2ca). **a** IKK-NF-kB pathways; and **b** inflammation and fibrosis. *N* = 6 per group, mean ± SE. *p < 0.05 versus wild-type mice

It is noteworthy that the data on mRNA expression is based on total RNA extracted from whole liver and may be influenced by non-hepatocyte gene expression. The Ikk2ca protein is Flag-tagged [9]. Hepatic mRNA expression of the Ikk2ca transgene was determined using forward and reverse primers that specifically target the Flag tag and Ikk2 cDNA, respectively. Ikk2ca mRNA was very low in the wild-type (Ikk2ca fl/+, Alb-cre/-) livers (Mean Ct value ~30), whereas the Ikk2ca mRNA expression levels in Liv-Ikk2ca mice (mean Ct value 24.7) appeared comparable to the expression levels of endogenous Ikk2 mRNA in wild-type mice (mean Ct value 24.3), estimated by the comparable Ct values of the Ikk2ca and mouse Ikk2 in these mice. Thus, hepatic expression of the Ikk2ca mRNA in the current Liv-Ikk2ca mice is likely lower than the two previous Liv-Ikk2ca mouse models in which the Ikk2ca transgene was driven by strong promoters from albumin and liver activator protein [5, 6]. Expectedly, hepatic mRNA expression of Ikk2 and classical targets of NF-kB activation, namely IkBα, serum amyloid a1 (Saa1), intercellular adhesion molecule 1 (Icam1), Bcl-x, and Akt1 [1, 3] were all significantly higher in 12-week-old male Liv-Ikk2ca mice than wild-type littermates (Fig. 1a). Conversely, Liv-Ikk2ca mice had comparable Gadd45b, RelA/p65, and Nfkb1 (p50), but moderately higher Ikk1 and much higher (6.9 fold) RelB (Fig. 1a). Interestingly, Liv-Ikk2ca mice had much higher (5.5 fold) Ikbke and 95 % higher NF-kB inducing kinase (NIK) which activates IKK1 [16] (Fig. 1a). Moreover, Liv-Ikk2ca mice had markedly higher cytokines lipocalin-2 (Lcn2, 106 fold) and A20 (9.4 fold) (Fig. 1a). Lcn2, an antimicrobial protein, protects endotoxin-induced sepsis [17] and diet-induced insulin resistance [18]. A20, an early NF-kB-responsive gene, has anti-inflammatory effects via negative feedback regulation of the classical NF-kB pathway [19] but activation of the alternative NF-kB pathway [20].

Surprisingly, markers of inflammation and fibrosis, namely Tnfα, IL-1β, IL-6, chemokine (C-C motif) ligand 2/monocyte chemotactic protein 1 (Ccl2/Mcp-1), Ccl5, Ccl19, Ccl20, NO synthase 2 (Nos2), transforming growth factor β (Tgfβ), α-smooth muscle actin (α-Sma), and collagen 1a1 (Col1a1), all remained unchanged in Liv-Ikk2ca mice (Fig. 1b), which is in a sharp contrast to marked induction of these genes as well as inflammation and fibrosis in adult mice with neonatal hepatocyte-specific activation of IKK2 [5]. In a previous study when hepatic expression of Ikk2ca was driven by the albumin promoter, although hepatic mRNA expression of IL-1β and IL-6 were strongly increased by 2.7 and 7.8 fold, respectively, blood levels of IL-1β remained unchanged whereas blood levels of IL-6 only doubled in those mice [6]. Consistent with the lack of increase in hepatic mRNA expression of inflammatory cytokines/chemokines, blood levels of IL-6 remained below the detection limit in wild-type and Liv-Ikk2ca mice, and there was no difference in blood levels of Mcp-1 between wild-type (96 ± 12 pg/ml) and Liv-Ikk2ca mice (67 ± 8 pg/ml).

### Liver histopathology in Liv-Ikk2ca mice

**Fig. 2:**
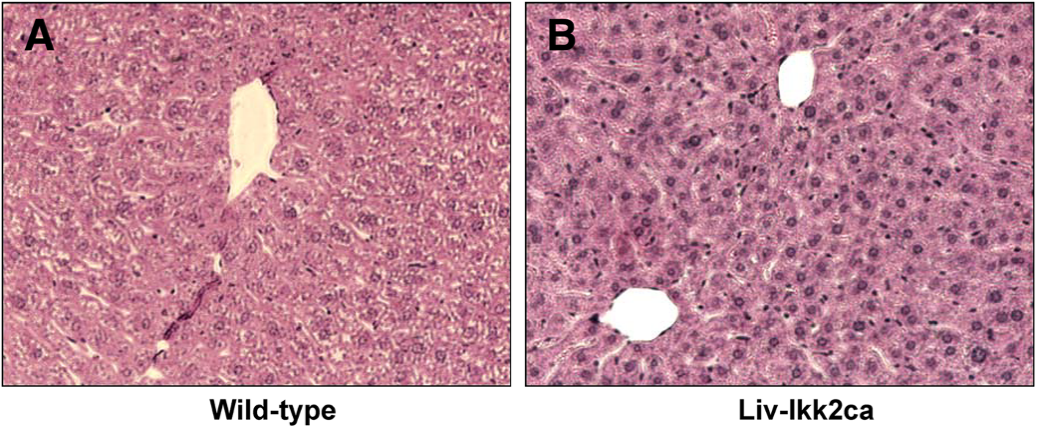
Liver histopathology in adult male mice with hepatocyte-specific activation of Ikk2 (Liv-Ikk2ca). **a** wild-type mice; and **b** Liv-Ikk2ca mice. Hematoxylin and eosin (H&E) staining of paraffin embedded liver sections (5 μm and 200 x magnification)

Histologically, the livers of wild-type (Fig. 2a) and Liv-Ikk2ca (Fig. 2b) mice were undistinguishable. There were no signs of infiltration of inflammatory cells or fibrosis in livers of Liv-Ikk2ca mice (Fig. 2b), which is consistent with the lack of induction of proinflammatory and fibrotic genes in these mice (Fig. 1).

### Hepatic mRNA expression of key antioxidative genes in Liv-Ikk2ca mice

**Fig. 3:**
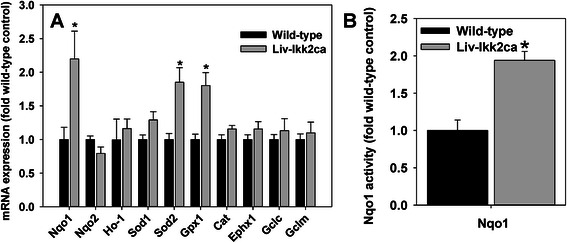
Hepatic mRNA expression of key antioxidative genes and Nqo1 activities in adult male mice with hepatocyte-specific activation of Ikk2 (Liv-Ikk2ca). **a** mRNA expression of antioxidative genes; and **b** Nqo1 activities. *N* = 6 per group, mean ± SE. *p < 0.05 versus wild-type mice

Liv-Ikk2ca mice had 120, 85, and 80 % higher expression of antioxidative genes Nqo1, superoxide dismutase 2 (Sod2), and glutathione peroxidase 1 (Gpx1), respectively (Fig. 3a). Conversely, Liv-Ikk2ca mice had similar Sod1, Nqo2, catalase (Cat), heme oxygenase-1 (Ho-1), epoxide hydrolase 1 (Ephx1), as well as glutamate-cysteine ligase catalytic subunit (Gclc) and modifier subunit (Gclm), key enzymes of glutathione synthesis.

To determine whether changes in mRNA expression translated into changes in protein expression and function, we determined Nqo1 activities in liver homogenates. The 1.2 fold higher mRNA expression of Nqo1 (Fig. 3a) was associated with similarly 94 % higher activities of Nqo1 in livers of Liv-Ikk2ca mice than wild-type mice (Fig. 3b).

### Hepatic mRNA expression of genes important in metabolism of glucose, lipids, cholesterol, and bile acids (BAs) in Liv-Ikk2ca mice

**Fig. 4:**
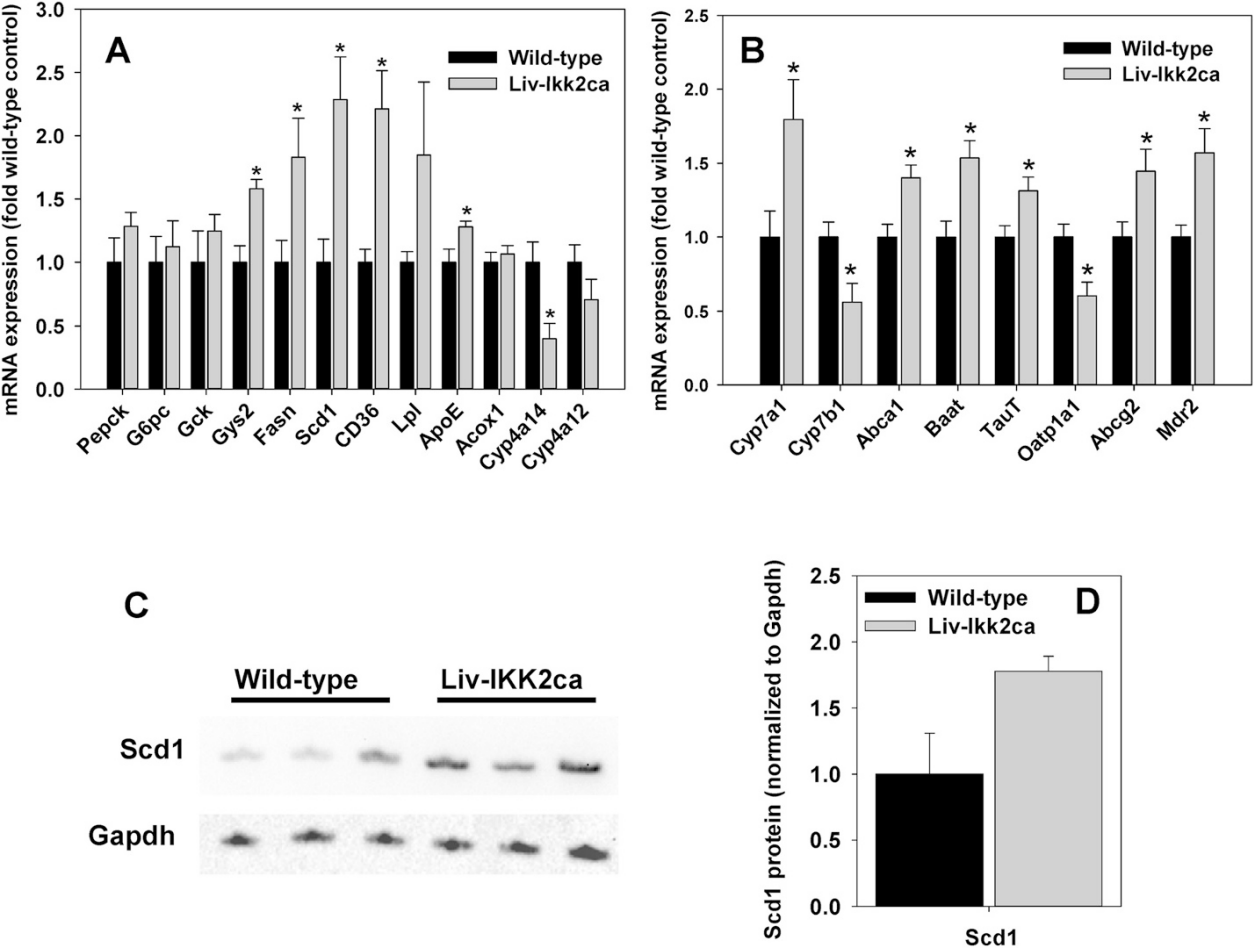
Hepatic expression of genes important in metabolism of glucose and lipids as well as cholesterol and bile acids in adult male mice with hepatocyte-specific activation of Ikk2 (Liv-Ikk2ca). **a** mRNA expression of genes in glucose and lipid metabolism; **b** mRNA expression of genes in cholesterol and bile acid metabolism. *N* = 6 per group, mean ± SE. *p < 0.05 versus wild-type mice. **c** & **d** Hepatic protein levels of Scd1 in adult male Liv-Ikk2ca mice. The densities of Scd1 in the gel image (top) were normalized to Gapdh (bottom). *N* = 3 per group, mean ± SE

Surprisingly, Liv-Ikk2ca mice and wild-type mice had similar expression of key enzymes for gluconeogenesis and glucose utilization, namely Pepck, G6pc, and glucokinase (Gck) (Fig. 4a). Conversely, Liv-Ikk2ca mice had 58 % higher glycogen synthase 2 (Gys2), a key enzyme for glycogen synthesis. Therefore, IKK2 activation in normal adult hepatocytes appears to produce a cytoprotective gene expression profile without apparent signs of inflammation, induction of gluconeogenic genes, or fibrosis.

Liv-Ikk2ca mice have 83 % higher fatty acid synthase (Fasn) and 1.2-fold higher CD36, a key uptake transporter of fatty acids (Fig. 4a). Stearoyl-CoA desaturase 1 (Scd1) is rate-limiting for the biosynthesis of monounsaturated fatty acids, whereas apolipoprotein E (ApoE) is essential for normal catabolism of triglyceride-rich lipoproteins. Liv-Ikk2ca mice had 120 and 28 % higher Scd1 and ApoE, respectively. Additionally, Liv-Ikk2ca mice had similar acyl-CoA oxidase 1 (Acox1), a key enzyme in peroxisomal fatty acid oxidation, but 60 % lower cytochrome P450 4a14 (Cyp4a14) and a trend of lower Cyp4a12, enzymes for microsomal fatty acid oxidation (Fig. 4a). Thus, compared to wild-type mice, livers of Liv-Ikk2ca mice had higher expression of genes important for the uptake (CD36) and synthesis (Fasn & Scd1) of fatty acids, but lower expression of genes for microsomal fatty acid oxidation (Cyp4a14).

Cyp7a1 and Cyp8b1 are two key enzymes in the classic pathway, whereas Cyp27a1 and Cyp7b1 are two key enzymes in the alternative pathway of BA biosynthesis from cholesterol. Liv-Ikk2ca mice had 80 % higher Cyp7a1 and 44 % lower Cyp7b1 (Fig. 4b), but similar Cyp8b1 and Cyp27a1 (Additional file 2). Liv-Ikk2ca mice had 40 % higher Abca1, which pumps cholesterol/phospholipids into circulation. Additionally, Liv-Ikk2ca mice had 54 % higher BA CoA: amino acid N-acyltransferase (Baat), a key enzyme for BA conjugation, and 31 % higher Taurine transporter (TauT). Therefore, compared to wild-type mice, livers of Liv-Ikk2ca mice had higher mRNA expression of the key genes for BA biosynthesis (Cyp7a1) and BA conjugation (Baat and TauT).

Liv-Ikk2ca mice had 40 % lower expression of uptake transporter organic anion-transporting polypeptide 1a1 (Oatp1a1) but 45 % higher efflux transporter Abcg2 and 57 % higher multi-drug resistance 2 (Mdr2), the biliary efflux transporter for phospholipids (Fig. 4b). Conversely, Liv-Ikk2ca mice and wild-type mice had comparable mRNA expression of other major BA uptake and efflux transporters (Additional file 2). In summary, Liv-Ikk2ca mice may have increased classic pathway of BA biosynthesis (by Cyp7a1), higher capacity for BA conjugation (by Baat and TauT), and higher biliary output of BAs (by Mdr2 and Abcg2).

To determine whether changes in mRNA expression translated into changes in protein expression, we used Western blot to determine Scd1 protein levels in liver homogenates. The 1.2 fold higher mRNA expression of Scd1 (Fig. 4a) was associated with similarly 78 % higher Scd1 protein in livers of Liv-Ikk2ca mice than wild-type mice (Fig. 4c & d).

### Hepatic and circulating levels of triglycerides and cholesterol in Liv-Ikk2ca mice

**Fig. 5:**
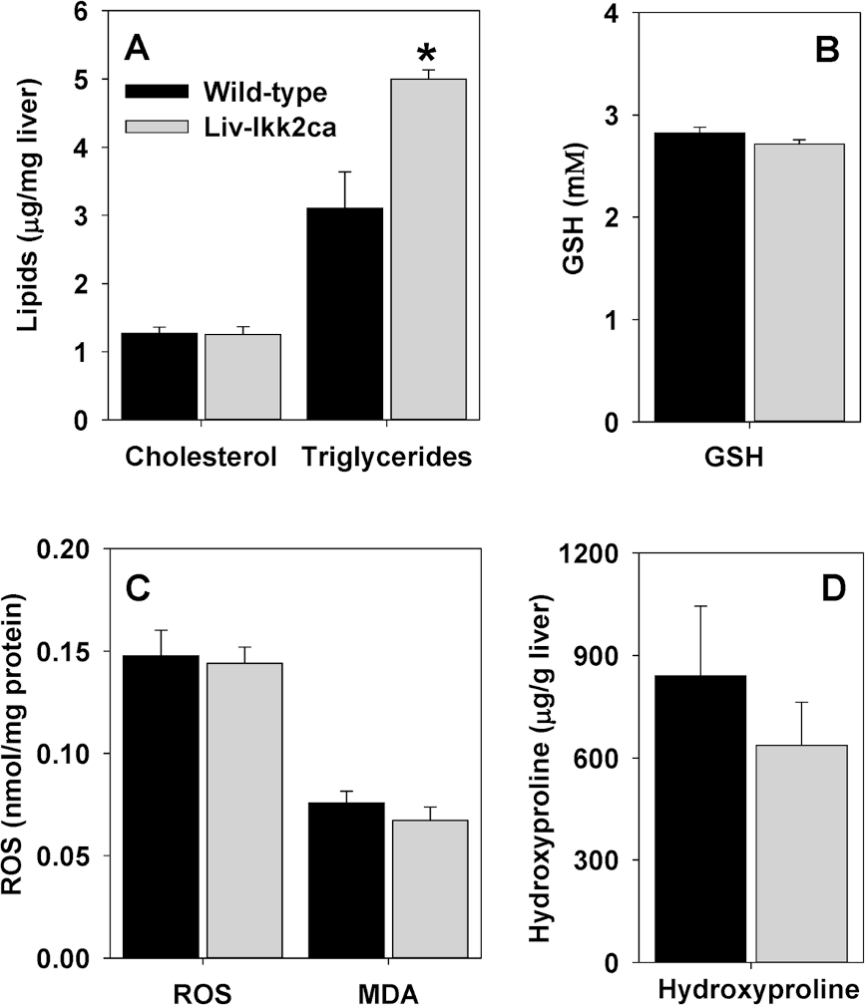
Hepatic levels of triglycerides, cholesterol, reduced GSH, endogenous reactive oxygen species (ROS), malondialdehyde (MDA), and hydroxyproline in adult male mice with hepatocyte-specific activation of Ikk2 (Liv-Ikk2ca). **a** triglycerides and cholesterol; **b** reduced GSH; **c** endogenous ROS and MDA; and **d** hydroxyproline. *N* = 6 per group, mean ± SE. *p < 0.05 versus wild-type mice

Consistent with higher hepatic expression of lipogenic genes, hepatic triglycerides were 61 % higher in Liv-Ikk2ca mice than in wild-type mice (Fig. 5a). Conversely, Liv-Ikk2ca mice had comparable cholesterol in liver (Fig. 5a) and similar serum levels of total cholesterol and triglycerides (data not shown).

### Hepatic levels of GSH, endogenous ROS, lipid peroxidation, and hydroxyproline in Liv-Ikk2ca mice

Activation of NF-kB has been associated with oxidative stress and steatohepatitis. However, we found that despite moderately elevated hepatic triglycerides in Liv-Ikk2ca mice, hepatic levels of GSH, endogenous ROS, and lipid peroxidation (MDA) remained unchanged in Liv-Ikk2ca mice compared to wild-type mice (Fig. 5b & c). Additionally, consistent with a lack of induction of fibrogenic genes, hepatic levels of hydroxyproline, a marker of fibrosis, remained unchanged in Liv-Ikk2ca mice (Fig. 5d).

### Hepatic mRNA expression of major drug-metabolizing enzymes in Liv-Ikk2ca mice

**Fig. 6:**
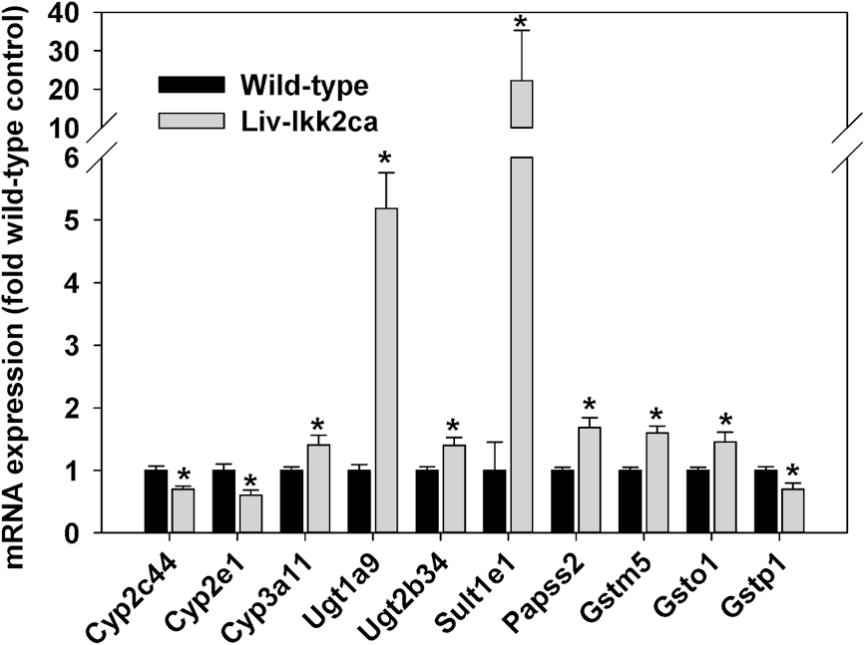
Hepatic mRNA expression of major cytochome P450s (Cyps) and Phase-II enzymes in adult male mice with hepatocyte-specific activation of Ikk2 (Liv-Ikk2ca). *N* = 6 per group, mean ± SE. *p < 0.05 versus wild-type mice

Activation of NF-kB has been implicated in down-regulation of drug-processing genes (DPGs) during inflammation [21]. Thus, we determined hepatic mRNA expression of major DPGs. Liv-Ikk2ca mice and wild-type mice had similar expression of most major Cyps, (Additional file 3A). However, Liv-Ikk2ca mice had 30 and 40 % lower Cyp2c44 and Cyp2e1, respectively, but 41 % higher Cyp3a11 (Fig. 6), the most predominant Cyp3a isoform in mouse liver [22].

Liv-Ikk2ca mice and wild-type mice had similar expression of most major UDP-glucuronosyltransferases (Ugts) and sulfotransferases (Sults) (Additional file 3B). Conversely, Liv-Ikk2ca mice had 4.2 fold, 40 %, and 21 fold higher Ugt1a9, Ugt2b34, and Sult1e1, respectively, and 69 % higher 3′-phosphoadenosine 5′-phosphosulfate synthetase 2 (Papss2) (Fig. 6), the major enzyme for PAPS biosynthesis in liver [23].

Liv-Ikk2ca mice and wild-type mice had similar expression of most major hepatic glutathione *S*-transferases (Gsts) [23], (Additional file 3C). Conversely, Liv-Ikk2ca mice had 60 % higher Gstm5 and 46 % higher Gsto1, but 30 % lower Gstp1 (Fig. 6). Thus, IKK2 activation in adult mouse hepatocytes does not down-regulate most major DPGs.

### Hepatic expression of essential transcription factors in Liv-Ikk2ca mice

**Fig. 7:**
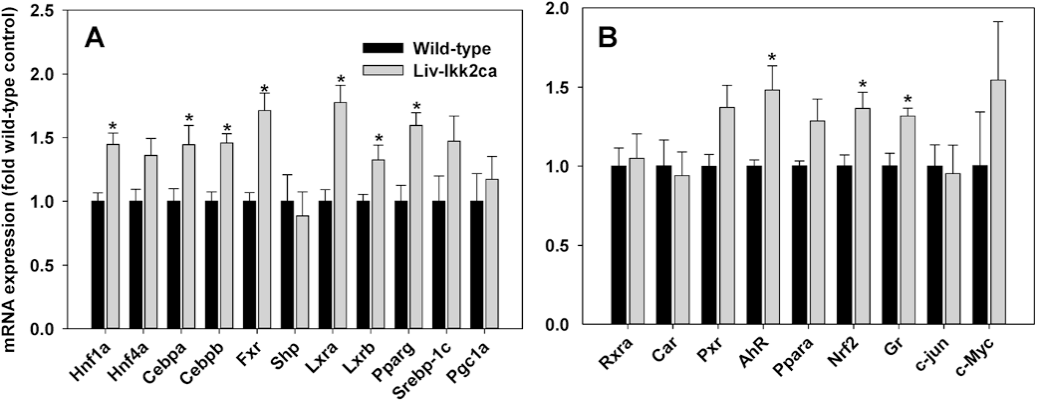
Hepatic mRNA expression of key transcription factors in adult male mice with hepatocyte-specific activation of Ikk2 (Liv-Ikk2ca). *N* = 6, mean ± SE. * *p* < 0.05 versus wild-type mice

**Fig. 8:**
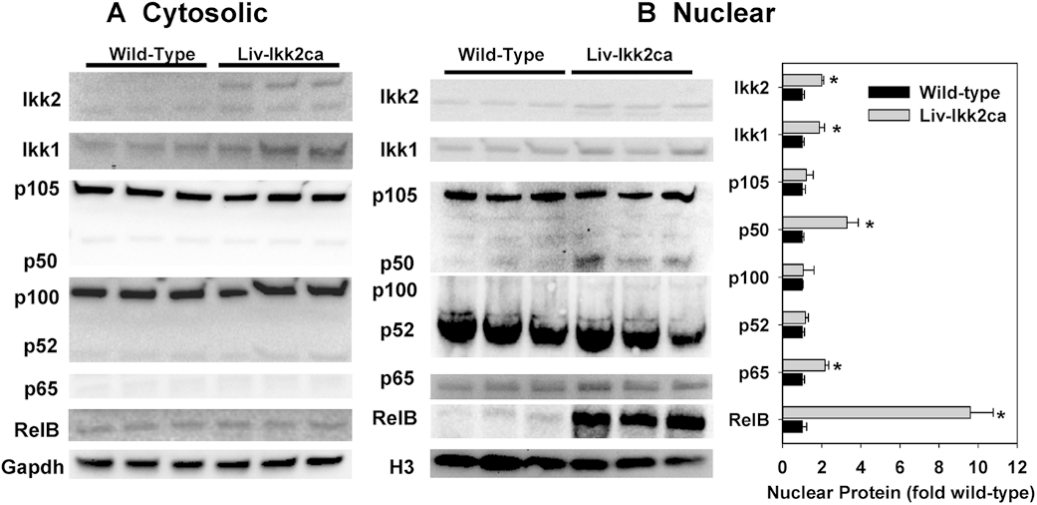
Western blot quantification of cytosolic and nuclear protein levels of Ikk and NF-kB subunits in adult male mice with hepatocyte-specific activation of Ikk2 (Liv-Ikk2ca). **a** cytosol; **b** nuclei. The densities of Ikk and each NF-kB subunit in the gel image (top) were normalized to Gapdh (**a**) or histone H3 (**b**). *N* = 3 per group, mean ± SE. *p < 0.05 versus wild-type mice

Transcription factors hepatocyte nuclear factor 1α (HNF1α), HNF4α, C/EBPα, C/EBPβ, farnesoid X receptor (FXR), liver X receptor α (LXRα), LXRβ, and peroxisome proliferator-activated receptor γ (PPARγ) as well as the co-activator PPARγ coactivator 1α (PGC1α) and the co-inhibitor small heterodimer partner (SHP) are essential for hepatic basal expression of genes important for nutrient and xenobiotic metabolism [24, 25]. Many of them were modestly higher in Liv-Ikk2ca mice, such as Hnf1a (45 %), Cebpa (44 %), Cebpb (46 %), and Fxr (71 %) (Fig. 8a). Interestingly, Liv-Ikk2ca mice had 59, 78, and 32 % higher expression of 3 lipogenic nuclear receptors, namely Pparγ, Lxrα, Lxrβ, respectively, and tended to have higher (47 %) lipogenic transcription factor Srebp-1c (Fig. 7a).

Xenobiotic receptors, namely aryl hydrocarbon receptor (AhR), constitutive active receptor (CAR), Pregnane X receptor (PXR), PPARα, and nuclear factor (erythroid-derived 2)-like 2 (NRF2) are very important in hepatic induction of DPGs by xenobiotics, and retinoid X receptor α (RXRα) is the obligatory heterodimer partner for FXR, CAR, PXR, and PPARs [26]. Glucocorticoid receptor (GR), c-Jun, and c-Myc are important in inflammation and stress responses. Liv-Ikk2ca mice had 48, 36, and 32 % higher AhR, Nrf2, and Gr, respectively, but similar Rxra, Car, Pxr, c-jun, and c-Myc (Fig. 7b).

Because different NF-kB subunits have differential effects on gene expression, we used Western blot to quantify hepatic nuclear and cytosolic protein levels of Ikk2, Ikk1, and major NF-kB subunits (Fig. 8). The Ikk2ca protein was Flag-tagged. Interestingly, an additional band was detected by Ikk2 antibody only in the cytosol of Liv-Ikk2ca mice, which might be the Ikk2ca protein and/or post-translationally modified Ikk2 protein (Fig. 8a, upper band). Nuclear levels of Ikk2 and Ikk1 were 100 and 88 % higher in Liv-Ikk2ca mice than wild-type mice, respectively (Fig. 8b). Nuclear levels of p65, p50, and RelB were 1.2-, 2.3-, and 8.6-fold higher in Liv-Ikk2ca mice (Fig. 8b), whereas p65, p50 and p52 were hardly detectable in the cytosol of both Liv-Ikk2ca mice and wild-type mice (Fig. 8a). Conversely, nuclear and cytosolic levels of precursors p100 and p105 remained unchanged (Fig. 8), and nuclear Foxo1, a transcription factor important in the regulation of insulin signaling and inflammation [27, 28] was very low in both strains (data not shown). Interestingly, p105, but not p100, was clearly present in the nuclei of both strains (Fig. 8b), whereas p52 appeared to be present at much higher levels than p50 in the nuclei in wild-type livers (Fig. 8b), which is consistent with high hepatic basal activity of Ikk1 [29].

### Binding of NF-kB p50 to the promoter of genes altered in livers of Liv-Ikk2ca mice

**Fig. 9:**
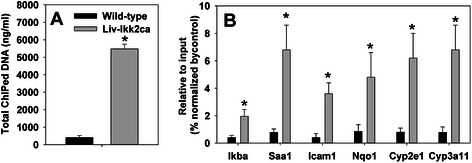
ChIP-qPCR determination of binding of NF-kB p50 to proximal promoters of genes altered in livers of adult male mice with hepatocyte-specific activation of Ikk2 (Liv-Ikk2ca). **a** total amount of ChIPed DNA; **b** qPCR quantification of enrichment of DNA fragments that contain putative NF-kB binding site. *N* = 3 per group, mean ± SE. *p < 0.05 versus wild-type mice

To determine whether increases in the nuclear NF-kB subunits (Fig. 8b) translate into increases the DNA-binding of NF-kB, we used ChIP-qPCR to determine DNA-binding of p50 in livers of Liv-Ikk2ca mice. The total amount of DNA fragments pulled down by the p50 antibody was 12.4 fold higher in Liv-Ikk2ca mice than wild-type mice (Fig. 9a). Additionally, results of ChIP-qPCR showed that the binding of p50 to the proximal promoters of Ikba, Saa1, Icam1, Nqo1, Cyp2e1, and Cyp3a11 were 5–9 fold higher in Liv-Ikk2ca mice than wild-type mice (Fig. 9b).

## Discussion

Two recent studies demonstrate that neonatal hepatocyte-specific superactivation of IKK2-NF-kB causes progressive inflammation and liver fibrosis at adulthood [5], whereas hepatocyte-specific activation of IKK2-NF-kB by transgenic expression of Ikk2ca driven by a strong albumin promoter causes inflammation and insulin resistance without liver fibrosis [6]. The present study demonstrates that hepatocyte-specific expression of Ikk2ca driven by a moderate endogenous Rosa26 promoter produces a cytoprotective gene expression profile, without apparent signs of hepatic inflammation or fibrosis in mice. However, these Liv-Ikk2ca mice have moderate increase in lipogenesis. Currently, it remains unknown the underlying mechanism of the marked differences in gene expression and liver pathophysiology between our Liv-Ikk2ca mice and the two previous mouse models [5, 6]. In all these three models, mice with liver-specific activation of Ikk2 were examined at around 12 weeks of age, and thus the differences in hepatic gene expression and pathophysiology among these mouse models are unlikely due to differences in age. Compared to the two previous mouse models [5, 6], our Liv-Ikk2ca mice do not have higher expression of many cytokines (e.g. TNFα, IL-1β, and IL-6) or chemokines (Mcp-1/Ccl2, Ccl5, and Ccl20), suggesting that these 3 Ikk2ca mouse models have significant difference(s) in the activation of the upstream transactivator(s) of these cytokines and chemokines.

A major potential mechanism of differences in hepatic gene expression and pathophysiology between the current mouse model and the two previous mouse models is differences in the magnitude of activation of IKK2-NF-kB pathway. In fact, the gene-dosage effect of Ikk2 activation on liver pathophysiology has been demonstrated in the study of mice in which the Ikk2ca transgene is driven by a strong albumin promoter [6]. Glucose intolerance and insulin resistance are more severe in the homozygous transgenic mice (with two alleles of Ikk2ca transgene) than the hemizygous transgenic mice [6]. In the model of neonatal hepatocyte-specific activation of IKK2-NF-kB, the IKK2ca protein is highly expressed in the transgenic liver, at levels much higher than the control mice [5]. In contrast, in the mouse model that hepatic expression of Ikk2ca is driven by an albumin promoter, hepatic IKK2ca protein was only moderately increased, judged from the Western blot data [6]. In the present study, hepatic expression of Ikk2ca is driven by the endogenous Rosa26 promoter [9], and hepatic mRNA and protein expression of Ikk2 in Liv-Ikk2ca mice are only moderately higher than wild-type mice (Fig. 8), which will translate into a moderate activation of the Ikk2-NF-kB pathway in mouse liver. Because of the putative large differences in the magnitude of hepatic expression of Ikk2ca protein, results from the previous two Liv-Ikk2ca mouse models (using promoters much stronger than the endogenous Ikk2 promoter) may reflect superactivation of Ikk2-NF-kB under certain pathological conditions [5, 6], whereas results from the current Liv-Ikk2ca mouse model (using a moderate promoter weaker than the endogenous Ikk2 promoter) may reflect physiological activation of Ikk2 under certain pathophysiological conditions.

The high basal nuclear p52 and strong induction of RelB (Fig. 8b) indicate that the IKK1-regulated alternative NF-kB pathway via RelB/p52 is strongly activated in livers of Liv-Ikk2ca mice. The higher nuclear p50 protein (Fig. 8b) without higher mRNA expression of Nfkb1 (Fig. 1a), the precursor of p50 in Liv-IKK2ca mice suggests a preferential activation of p50 over p65 in the classical NF-kB signaling pathway. In contrast, there is marked increase in nuclear translocation of p65 in adult livers of mice with neonatal activation of IKK2, which is associated with inflammation and fibrosis [5]. Consistently, mice with knockin of constitutively active p65 develop systemic inflammation and liver fibrosis [30]. The NF-kB p50 can form heterodimer with both p65 and RelB, and the p65/p50 and RelB/p50 heterodimers have distinct target genes and physiological functions. The RelB/p50 heterodimer inhibits TNFα expression [31]. Importantly, RelB and IKK1 are essential in anti-inflammation. RelB knockout mice develop spontaneous multiorgan inflammation [32], which is worsened by concomitant loss of p50 [33]. During endotoxin tolerance, induction of RelB is necessary and sufficient to silence proinflammatory genes [34]. IKK1 is constitutively active in unstimulated adult hepatocytes [29], which is consistent with the high nuclear levels of p52 in wild-type livers (Fig. 8b). Loss of IKK1 prolongs the activation of IKK2 and promotes inflammation [35]. IKK1 accelerates the removal of RelA/p50 subunits from proinflammatory gene promoters to limit NF-kB activation [35]. Importantly, in unstimulated cells, assembly of IKK1 with IKK2 into a heterodimeric complex inhibits the high intrinsic activity of IKK2 [36].

It is well established that NF-kB activity is very low in unstressed adult liver [37]; however, the underlying mechanism remains elusive. The p105 precursor of p50, encoded by Nfkb1, plays a key role in inhibition of NF-kB activation. Previous cellular study showed that p105 is exclusively located in the cytosol [38]. Interestingly, we found that p105 is also readily detectable in the nuclei of unstressed adult liver (Fig. 8b). Thus, the high basal activities of Ikk1/p52 and nuclear p105 may help maintain the basal activities of NF-kB at low levels in unstressed adult liver, and the further induction and activation of Ikk1 and RelB after a moderate activation of Ikk2 by the Ikk2ca protein may play a key role in preventing the overactivation of IKK2/NF-kB and resultant induction of proinflammatory genes (e.g. IL-6 and Mcp-1) in Liv-Ikk2ca mice.

**Fig. 10:**
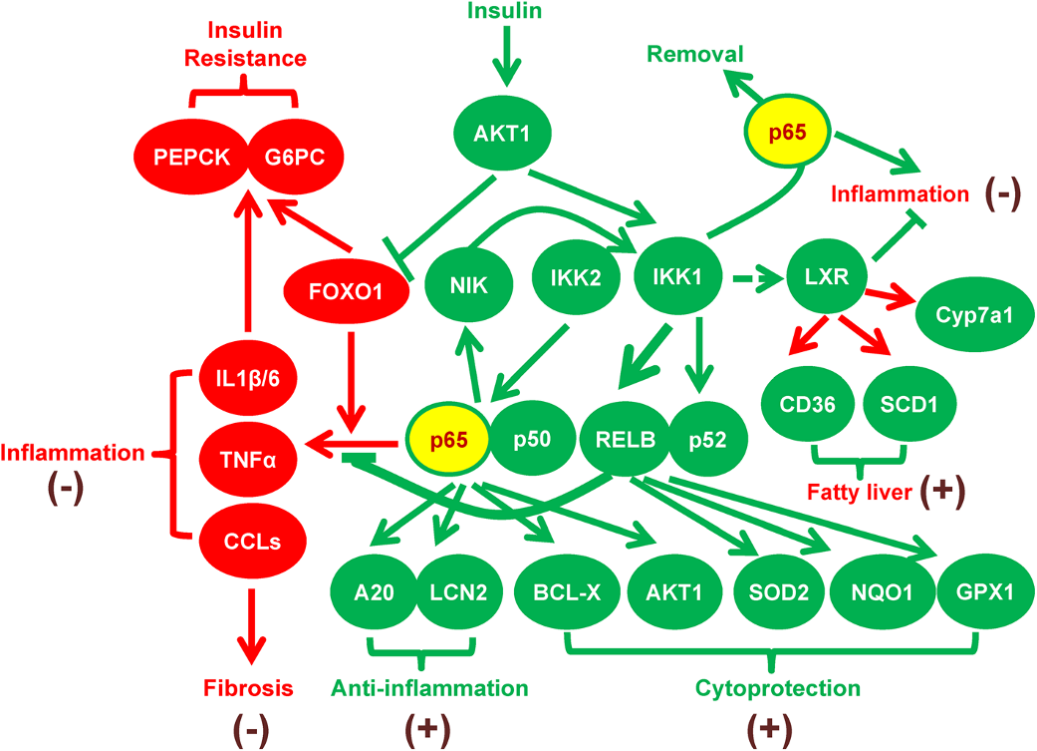
Diagram that summarizes the possible underlying mechanisms of key changes in gene expression and pathophysiology in adult male mice with hepatocyte-specific activation of Ikk2 (Liv-Ikk2ca). Genes that are active or induced in Liv-Ikk2ca mice are in green shape, whereas genes that are inactive or unchanged in Liv-Ikk2ca mice are in red shape. Pathways/responses that are activated in Liv-Ikk2ca mice are marked with (+), whereas pathways/responses that are inhibited or unchanged in Liv-Ikk2ca mice are marked with (−)

FOXO1 inhibits insulin sensitivity, and FOXO1 synergizes with NF-kB p65 and/or p50 to induce proinflammatory IL-1β, IL-6, and CCL20 [28, 39, 40], genes induced in adult mice with neonatal liver-specific activation of Ikk2 [5] but not in the present Liv-Ikk2ca mice (Fig. 1). Importantly, FOXO1 dramatically induces CCL20 in an NF-kB-dependent manner in HepG2 cells [39]. Moreover, FOXO1 induces CCL2 and IL-6 [41]. FOXO1 protein is abundantly present in the nucleus of neonatal liver, but only at very small amount in the nucleus of adult liver [42]. Additionally, FOXO1 is markedly activated during fasting [43], a condition used for the study of glucose intolerance and insulin resistance [6]. The Liv-IKK2ca mice were not fasted before euthanasia in this study. Taken together, we propose a novel hypothesis that the magnitude of IKK2 activation, through interaction with FOXO1 and IKK1, play key roles in determining the protective versus detrimental effect of IKK2-NF-kB activation in liver. When IKK2 is activated moderately in normal adult liver with normal insulin signaling, active IKK1 balances the NF-kB signaling by activating p52/RelB but preventing overactivation of p65, and the low FOXO1 activity also prevents induction of proinflammatory cytokines/chemokines, resulting in induction of cytoprotective genes without inflammation and fibrosis, as observed in the present study (Fig. 10). Conversely, when IKK2 is superactivated in fasting liver or neonatal liver, where IKK1 is low and FOXO1 activity is high [42], FOXO1 synergizes with p65/p50 to cause insulin resistance and induce proinflammatory IL-1β, IL-6, CCL2, and CCL20, resulting in progressive inflammation and/or liver fibrosis [5].

It is noteworthy that Ikk2 has NF-kB-independent functions in immunity. Ikk2 can phosphorylate tuberous sclerosis 1, resulting in activation of the mTOR pathway [44]. mTOR has multifunctional role in inflammation; inhibition of mTOR causes distinct inflammatory side effects such as fever, pneumonitis, glomerulonephritis or anemia of chronic disease [45]. Thus, the NF-kB-independent function of IKK2, such as activation of the mTOR pathway, might suppress inflammation in the context of low expression of activated Ikk2.

Previous *in vitro* studies suggest that activation of NF-kB plays a key role in the down-regulation of DPGs during inflammation [46]. The present study demonstrates that moderate activation of IKK2-NF-kB by Ikk2ca does not down-regulate most major DPGs. Conversely, activation of NF-kB induces Cyp3a11 whose human ortholog CYP3A4 plays a key role in the metabolism of numerous drugs [47]. The down-regulation of DPGs by NF-kB *in vitro* is ascribed to direct binding of p65 to promoters and/or indirect inhibition of DPGs via inhibitory interactions of p65 with other key transcription factors such as HNF4α, GR, PXR, and CAR [46, 48]. In the present study, nuclear p65 is increased moderately whereas p50 and RelB are increased more markedly (Fig. 8b), and hepatic expression of transcription factors essential for DPG expression are either unaltered or induced in Liv-Ikk2ca mice (Fig. 7). Thus, activation of IKK2-NF-kB in the absence of overt inflammation does not down-regulate most DPGs; however, the role of superactivation of NF-kB p65 in down-regulation of DPGs during inflammation cannot be excluded.

In addition to inflammation, the IKK2-NF-kB pathway can be activated by diverse stress signaling such as oxidative stress and xenobiotic exposure, and activation of NF-kB often protects against oxidative stress [49]. Interestingly, our study provides the first evidence that moderate activation of IKK2 in normal adult mouse liver down-regulates Cyp2e1, Cyp4a14, and Gstp1 (Fig. 6), genes involved in generating oxidative stress and liver injury, but induces antioxidative genes Nqo1, Sod2, Gpx1 (Fig. 3a), and Abcg2 (Fig. 4b) as well as cytoprotective genes Bcl-xl and Akt1 (Fig. 1). Activation of NF-kB and/or induction of Nrf2 (Fig. 7) may be the underlying mechanism of induction of these cytoprotective genes. For example, Sod2 is a direct target of RelB [50], and Nqo1, Sod2, Gpx1, and ABCG2 are known NF-kB target genes in cellular studies [49, 51]. Cyp4a14 is dramatically induced in livers of Nrf2-null mice [12]; thus, the moderate induction of Nrf2 might play a role in the down-regulation of Cyp4a14 in Liv-Ikk2ca mice. CYP2E1 and CYP4A are the two major CYP enzymes that generate superoxide under pathophysiological conditions [52]. SOD2 protects against mitochondrial damage by converting superoxide to the less reactive H_2_O_2_, and GPX1 prevents many diseases by limiting H_2_O_2_ accumulation. NQO1 plays a key role in antioxidative response and metabolism of many toxicants and drugs [53]. ABCG2 prevents intracellular accumulation of porphyrins and oxidative injury [54]. Additionally, activation of IKK2-NF-kB causes induction of Baat (the sole enzyme for conjugation of BAs to taurine and glycine [55]) and Mdr2 (Fig. 4b), which are essential in the protection against cholestatic liver injury [56]. By coordinated down-regulation of prooxidative genes CYP2E1 and CYP4A but induction of cytoprotective genes SOD2, NQO1, GPX1, ABCG2, BAAT, and MDR2, moderate activation of IKK2-NF-kB in adult hepatocytes might exert hepatoprotective effects [3, 4].

The present study demonstrates that moderate activation of the IKK2-NF-kB in normal adult liver causes moderate increase of lipogenesis without overt inflammation, which is associated with induction of lipogenic transcription factors PPARγ, LXRα, and LXRβ [57] (Fig. 7) as well as lipogenic Fasn, Scd1, and CD36 (Fig. 4a). Thus, moderate activation of IKK2-NF-kB alone in unstressed adult hepatocytes is sufficient to cause increase of lipogenesis but not steatohepatitis. Liv-Ikk2ca mice have higher expression of a group of LXR-target genes, including Cd36, Scd1, Cyp7a1, ApoE, and Abca1 [57]. LXR is likely activated in Liv-Ikk2ca mice via induction of LXRs (Fig. 7a) and accumulation of its ligand, oxysterols due to down-regulation of Cyp7b1 (Fig. 4b), an oxysterol 7α-hydroxylase. Activation of LXR promotes lipogenesis but exerts anti-inflammatory effects [58]. Thus, activation of LXRs might contribute to the increase of lipogenesis without inflammation in Liv-Ikk2ca mice (Fig. 10).

Although non-alcoholic fatty liver disease (NAFLD) is highly prevalent in modern societies, only 10–25 % of NAFLD cases develop hepatic fibrosis leading to cirrhosis, end-stage liver disease or hepatocellular carcinoma [59]. NF-kB is activated by diverse stresses [3, 4], and activation of NF-kB plays an important role in the etiology of steatohepatitis [59]. However, activation of NF-kB also protects liver against injuries induced by inflammation and xenobiotics [3, 4], and hepatocyte-specific activation of NF-κB does not aggravate chemical hepatocarcinogenesis in transgenic mice [60]. The present study demonstrates that moderate activation of IKK2-NF-kB in unstressed adult liver produces a cytoprotective gene expression profile and increases lipogenesis without induction of gluconeogenic genes or signs of inflammation and fibrosis. It is noteworthy that liver fibrosis only occurs in the mice with neonatal activation of Ikk2 and extensive inflammation [5], but not in the Ikk2ca transgenic mice with only mild inflammation [6]. Therefore, in future studies, it is very important to identify the key factor(s) that interacts with IKK2-NF-kB during pathological conditions and switches the function of IKK2-NF-kB from liver protection to pro-inflammation and pro-fibrosis, which might allow us to selectively modulate the IKK-NF-kB pathways to improve the efficacy and decrease the toxicity of drug treatment of metabolic syndrome, infectious/inflammatory diseases, and liver diseases.

## Conclusions

The present study demonstrates that moderate activation of IKK2-NF-kB in unstressed adult mouse liver produces a cytoprotective gene expression profile and increases lipogenesis without apparent signs of inflammation or fibrosis, likely due to strong activation of the anti-inflammatory IKK1-RelB alternative NF-kB pathway as well as activation of the Lxr.

## Additional files

Additional file 1: **List of primers used for ChIP-qPCR determination of DNA-binding of p50 in Liv-Ikk2ca mice.** Description of data: sequence of ChIP-qPCR primers. (XLS 21 kb)
Additional file 2: **Hepatic mRNA expression of major bile acid transporters in adult male mice with hepatocyte-specific activation of Ikk2 (Liv-Ikk2ca).** Description of data: mRNA expression. (PDF 14 kb)
Additional file 3: **Hepatic mRNA expression of major drug metabolizing enzymes in adult male mice with hepatocyte-specific activation of Ikk2 (Liv-Ikk2ca).** Description of data: mRNA expression. (PDF 20 kb)

## Abbreviations

IKK2ca: Constitutive active IKK2
Liv-Ikk2ca: Adult hepatocyte-specific activation of Ikk2
IKK1: Inhibitor of nuclear factor-kappaB (IkappaB) kinase 1
IkBα: Inhibitor of kB α
TNFα: Tumor necrosis factor α
IL-6: Interleukin-6
Pepck: Phospho-enolpyruvate carboxykinase
G6pc: Glucose-6-phosphatase, catalytic subunit
IACUC: Institutional Animal Care and Use Committee
Gapdh: Glyceraldehyde 3-phosphate dehydrogenase
Nqo1: NAD(P)H quinone oxidoreductase 1
TG: Triglycerides
CHO: Cholesterol
Saa1: Serum amyloid a1
Icam1: Intercellular adhesion molecule 1
NIK: NF-kB inducing kinase
Lcn2: Lipocalin-2
Ccl2: (C-C motif) ligand 2
Nos2: NO synthase 2
Tgfβ: Transforming growth factor β
α-Sma: α-smooth muscle actin
Col1a1: Collagen 1a1
Sod2: Superoxide dismutase 2
Gpx1: Glutathione peroxidase 1
Cat: Catalase
Ho-1: Heme oxygenase-1
Ephx1: Epoxide hydrolase 1
Gclc: Glutamate-cysteine ligase catalytic subunit
BAs: Bile acids
Gck: Glucokinase
Gys2: Glycogen synthase 2
Fasn: Fatty acid synthase
Lpl: Lipoprotein lipase
Scd1: Stearoyl-CoA desaturase 1
ApoE: Apolipoprotein E
Acox1: Acyl-CoA oxidase 1
Cyp4a14: Cytochrome P450 4a14
Baat: Bile acid
CoA: Amino acid N-acyltransferase
TauT: Taurine transporter
Oatp1a1: Organic anion-transporting polypeptide 1a1
Mdr2: Multi-drug resistance 2
DPGs: Drug-processing genes
Por: P450 oxidoreductase
Ugts: UDP-glucuronosyltransferases
Sults: Sulfotransferases
Ugp2: UDP-glucose pyrophosphorylase 2
Papss2: 3′-phosphoadenosine 5′-phosphosulfate synthetase 2
Gsts: Glutathione *S*-transferases
HNF1α: Hepatocyte nuclear factor 1α
FXR: Farnesoid X receptor
LXRα: Liver X receptor α
PPARγ: Peroxisome proliferator-activated receptor γ
PGC1α: PPARγ coactivator 1α
SHP: Small heterodimer partner
AhR: Aryl hydrocarbon receptor
CAR: Constitutive active receptor
PXR: Pregnane X receptor
NRF2: Nuclear factor (erythroid-derived 2)-like 2
RXRα: Retinoid X receptor α
GR: Glucocorticoid receptor
NAFLD: Non-alcoholic fatty liver disease
