# Supplementary material for: Moderate activation of IKK2-NF-kB in unstressed adult mouse liver induces cytoprotective genes and lipogenesis without apparent signs of inflammation or fibrosis
Source: BMC Gastroenterol. 2015 Jul 30;15:94. doi: 10.1186/s12876-015-0325-z (PMC4518658; doi:10.1186/s12876-015-0325-z)
Supplement: Additional file 3: — Hepatic mRNA expression of major drug metabolizing enzymes in adult male mice with hepatocyte-specific activation of Ikk2 (Liv-Ikk2ca). Description of data: mRNA expression. (PDF 20 kb) [file 12876_2015_325_MOESM3_ESM.pdf]

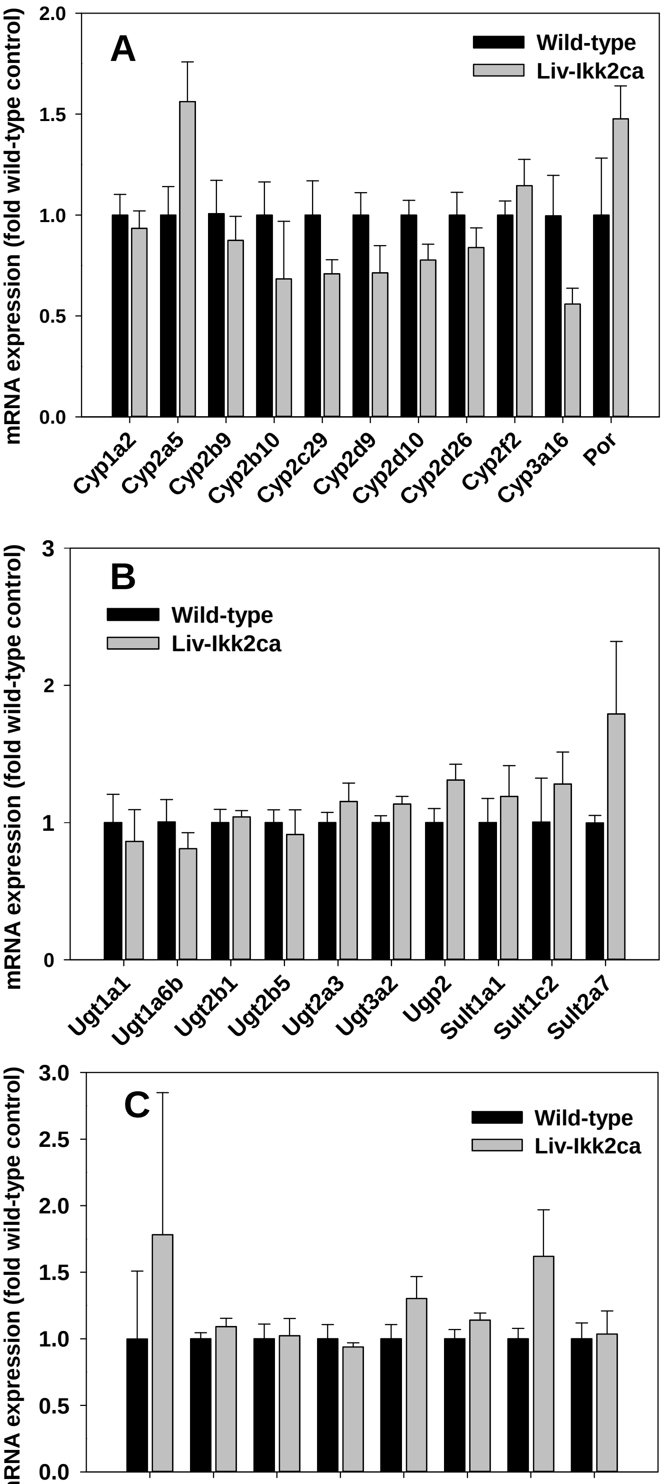

Additional file 3. Hepatic mRNA expression of major drug metabolizing enzymes in adult male mice with hepatocyte-specific activation of Ikk2 (Liv-Ikk2ca). N=6, mean ± SE.
